# Potential Measurement Errors Due to Image Enlargement in Optical Coherence Tomography Imaging

**DOI:** 10.1371/journal.pone.0128512

**Published:** 2015-05-29

**Authors:** Akihito Uji, Tomoaki Murakami, Yuki Muraoka, Yoshikatsu Hosoda, Shin Yoshitake, Yoko Dodo, Shigeta Arichika, Nagahisa Yoshimura

**Affiliations:** Department of Ophthalmology and Visual Sciences, Kyoto University Graduate School of Medicine, Kyoto 606–8507, Japan; Massachusetts Eye & Ear Infirmary, Harvard Medical School, UNITED STATES

## Abstract

The effect of interpolation and super-resolution (SR) algorithms on quantitative and qualitative assessments of enlarged optical coherence tomography (OCT) images was investigated in this report. Spectral-domain OCT images from 30 eyes in 30 consecutive patients with diabetic macular edema (DME) and 20 healthy eyes in 20 consecutive volunteers were analyzed. Original image (OR) resolution was reduced by a factor of four. Images were then magnified by a factor of four with and without application of one of the following algorithms: bilinear (BL), bicubic (BC), Lanczos3 (LA), and SR. Differences in peak signal-to-noise ratio (PSNR), retinal nerve fiber layer (RNFL) thickness, photoreceptor layer status, and parallelism (reflects the complexity of photoreceptor layer alterations) were analyzed in each image type. The order of PSNRs from highest to lowest was SR > LA > BC > BL > non-processed enlarged images (NONE). The PSNR was statistically different in all groups. The NONE, BC, and LA images resulted in significantly thicker RNFL measurements than the OR image. In eyes with DME, the photoreceptor layer, which was hardly identifiable in NONE images, became detectable with algorithm application. However, OCT photoreceptor parameters were still assessed as more undetectable than in OR images. Parallelism was not statistically different in OR and NONE images, but other image groups had significantly higher parallelism than OR images. Our results indicated that interpolation and SR algorithms increased OCT image resolution. However, qualitative and quantitative assessments were influenced by algorithm use. Additionally, each algorithm affected the assessments differently.

## Introduction

“Pixel,” the blended word for “picture” and “element,” is the smallest portion of a digital image. Therefore, digital image resolution is expressed as the product of the horizontal and vertical number of pixels.[1] When digital images are enlarged, each pixel is also enlarged to several times the original size, and no new information is gained. However, many image-editing software applications now utilize interpolation algorithms, which compute and insert new pixels to smooth the enlarged image and eliminate the characteristic saw-toothed outlines of digitally enlarged images. These new computed pixels can provide new information and enlarge images with the same capacity as optical enlargements, almost like examining objects through a magnifying glass. However, we should keep in mind that the newly obtained information did not exist in the original image.

Optical imaging technology has undergone amazing advancements in the past few decades. This is especially true for optical coherence tomography (OCT), which noninvasively provides microscopic cross-sectional images of the retina, and has become an indispensable tool in managing patients and understanding retinal disease.[2,3,4,5] Higher-resolution OCT images and digital smoothing techniques are increasingly allowing microscopic retinal details to be detected,[6,7,8,9,10,11] but this has brought about a new problem. Image enlargement through interpolation algorithms allows higher levels of qualitative and quantitative assessment of retinal details. However, investigators should pay careful attention to potential inaccuracies caused by pixel interpolation. For example, an OCT image with a longitudinal resolution of 5 μm/pixel will have its resolution increased to 1 μm/pixel when scaling up the digital image by a factor of five. Interpolation algorithms are useful in producing a clear enlarged image, but understanding algorithm limitations is extremely important.

All algorithms have their advantages and disadvantages. The bilinear (BL) and bicubic (BC) algorithms are basic resampling techniques and widely used algorithms in computer application software. The BC-interpolated surface is smoother than the corresponding BL-interpolated surface. Additionally, the Lancsoz3 (LA) algorithm produces a less blurred image than the BC algorithm, but has a slow performance. The super-resolution (SR) algorithm is a promising technology, which uses signal processing techniques to reconstruct high resolution images. In this study, we investigate the effect of interpolation (BL, BC, and LA) and SR algorithms[12] on the quantitative and qualitative assessment of enlarged OCT images.

## Methods

### Subjects

The present study had a retrospective, observational design for evaluating a diagnostic test. All study conduct adhered to the tenets of the Declaration of Helsinki and the study protocol was approved by the Institutional Review Board of Kyoto University Graduate School of Medicine. The Institutional Review Board waived the need for written informed consent from the participants. Data were retrospectively collected from the medical records of 20 eyes of 20 normal volunteers in the clinic database and from 30 eyes of 30 patients with diabetic macular edema (DME) who visited Kyoto University Hospital between February 2009 and March 2014. Patients were included if SD-OCT images of sufficient quality were available. Patients were excluded from analyses if there was evidence of serous retinal detachment, hard exudates at the fovea, or other retinal disease. Patient records were anonymized prior to analysis.

### Optical coherence tomography imaging

One horizontal line scan through the fovea, acquired with SD-OCT (Spectralis; Heidelberg Engineering, Heidelberg, Germany) was used for each eye included in this study. Images were originally acquired using thirty-degree cross-hair scans in the high resolution mode (1536 pixel x 496 pixel). We exported all images in a tagged image file format with an aspect ratio of 1.0 (pixel/pixel).

### Image analyses

#### Image preparation

**Fig 1:**
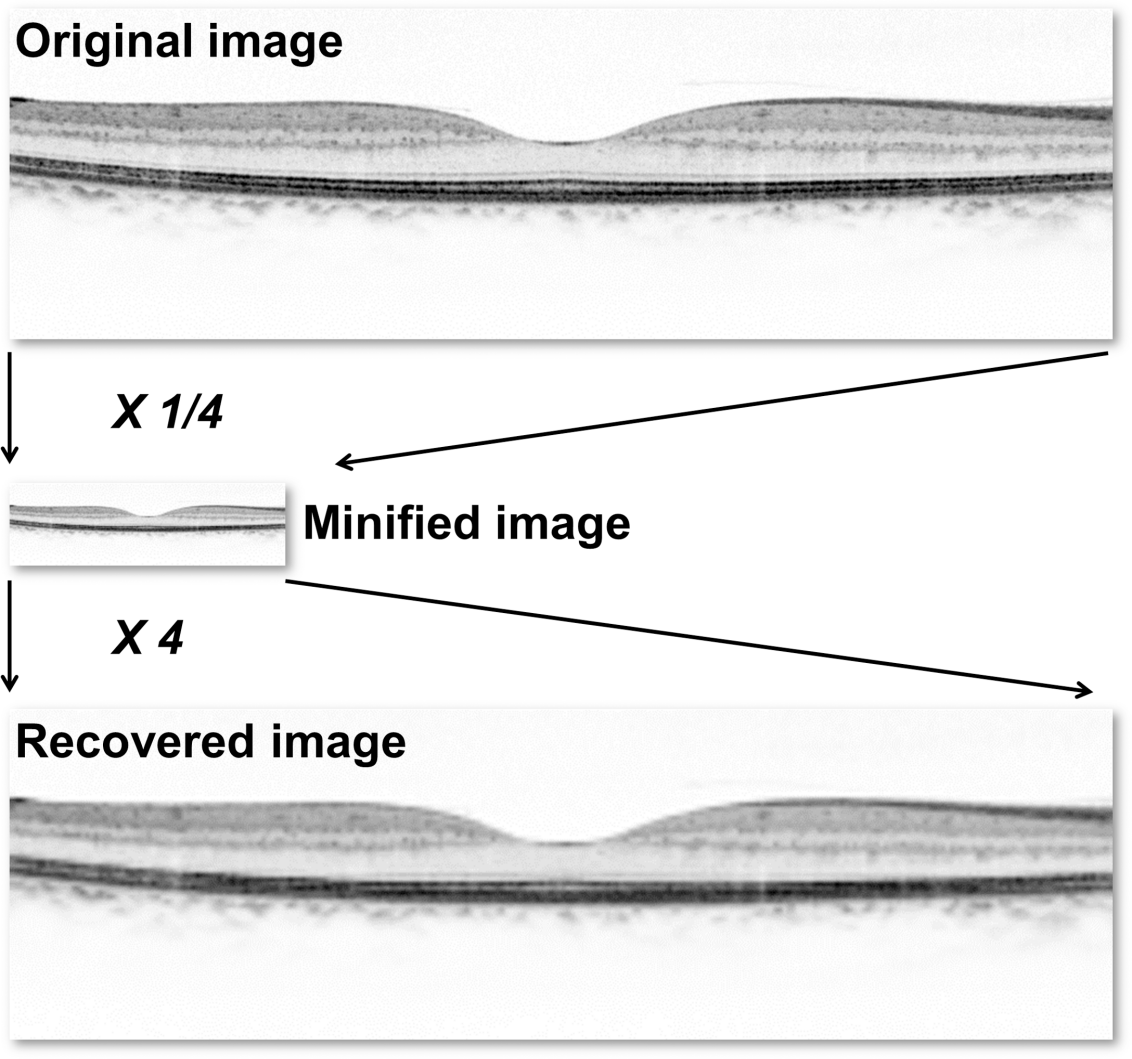
Preparation of optical coherence tomography images. Spectral-domain optical coherence tomography images were minified by a factor of four. They were then magnified by a factor of four to enlarge pixel size. Various interpolation and super-resolution algorithms were applied. The recovered image shown utilized the bicubic interpolation algorithm.

Because true high resolution OCT images, which should be used to compare enlarged images, did not exist, minified OCT images (Fig 1) were used to test smoothing algorithms.[13] All original images (OR) had their resolution reduced by a factor of four (minified). They were then magnified by a factor of four both with and without an interpolation (BL, BC,[14] or LA[15]) or an SR[12,16] algorithm. Therefore, six different types of images were used for analyses (i.e., OR, enlarged image without interpolation [NONE], BL, BC, LA, and SR). Image enlargement with the BL, BC, and LA algorithms was performed using MATLAB (Mathworks Inc., Natick, MA) and the MATLAB Image Processing Toolbox function. In this study, we used the Learning-Based SR algorithm, which uses a dictionary of many defined image patterns of correspondence to patch and convert a single-input, low resolution image to a high resolution image.[17,18] The SR100x100 software (http://cas.eedept.kobe-u.ac.jp/WelcomeES1/OpenSoft/SR100x100/index.html) was developed by Kuroki et al. and was used to apply the learning-based SR algorithm to enlarged images.

#### Peak signal-to-noise ratio measurement

Peak signal-to-noise ratio (PSNR) was measured to evaluate the image quality of the 5 different recovered OCT images.[19,20] The PSNR is generally used as a measure of recovered image degradation that occurs as a result of using enlarging algorithms. The PSNR is calculated by comparing the original and enlarged images, where the signal is original image data and noise is error caused by image enlargement. The higher the PSNR, the closer the enlarged image is to the original. A difference in PSNR of >0.2 dB is visible to the human eye. The PSNR was calculated in our study using ImageJ software (National Institutes of Health, Bethesda, MD; available at http://rsb.info.nih.gov/ij/index.html) and its plug-in software SNR (http://bigwww.epfl.ch/sage/soft/snr/).

#### Retinal nerve fiber layer thickness measurement

Retinal nerve fiber layer (RNFL) thickness was measured (in pixels) in normal subjects using both original and enlarged images. Differences between OR and enlarged images (NONE, BL, BC, LA, SR) and between enlarged images with (BL, BC, LA, SR) and without (NONE) interpolation algorithms were analyzed in each subject. The RNFL thickness was measured 0.5, 1.5, and 3.0 mm from the fovea. To evaluate interobserver correspondence of RNFL measurements, five images from each of the six different OCT image groups were randomly selected. Therefore, a total of 30 images were used from two independent examiners (retina specialists YM, YH).

#### Qualitative and quantitative photoreceptor layer assessment in eyes with diabetic macular edema

Differences in qualitative and quantitative photoreceptor layer assessments in eyes with DME were analyzed among the six different OCT image groups. For qualitative assessment, the photoreceptor layer status within 1 mm of the presumed fovea was categorized in each eye. Image examiners evaluated continuity of the external limiting membrane (ELM) and inner segment ellipsoid (ISe) lines, which were classified as complete, discontinuous, or absent.[10,21,22] To evaluate interobserver reproducibility, five images were randomly selected from the six different OCT image groups. Therefore, a total of 30 images were independently examined by 2 retina specialists (YM, YH). The kappa coefficient was calculated as a measure of agreement between the two observers.

The photoreceptor layer was also quantitatively assessed in eyes with DME. Parallelism, which we previously introduced as a new parameter of complexity of photoreceptor-retinal pigment epithelium complex alterations in eyes with DME, was determined within 1 mm of the presumed fovea.[23,24] Parallelism ranges between 0 and 1, with more complicated or destructively changed images having a higher parallelism.[25,26]

### Statistical analyses

All values are expressed as mean ± standard deviation. Comparisons of PSNR, RNFL thickness, and parallelism among the six different types of images were analyzed using the paired t-test, followed by Bonferroni correction. The chi-square test was used to test the statistical significance of differences in qualitatively assessed ISe and ELM status between original and recovered images. A *P* value of < 0.05 was considered to be statistically significant. All statistical analyses, with exception of the intraclass correlation coefficient (ICC) and the kappa coefficient, were performed using StatView (version 5.0, SAS Institute, Cary, NC). Calculation of the ICC and the kappa coefficient were performed using SPSS software (version 17, SPSS, Inc., Chicago, IL).

## Results

A total of 30 patients with DME (average age = 65.1 ± 5.7 years, range: 49–77 years) and 20 normal volunteers (average age = 63.8 ± 11.3 years, range: 38–77 years) were included in this study. This allowed us to examine how image-enhancing algorithms affect OCT images from both the normal and diseased retina.

### Peak signal-to-noise ratio in recovered OCT images

**Fig 2:**
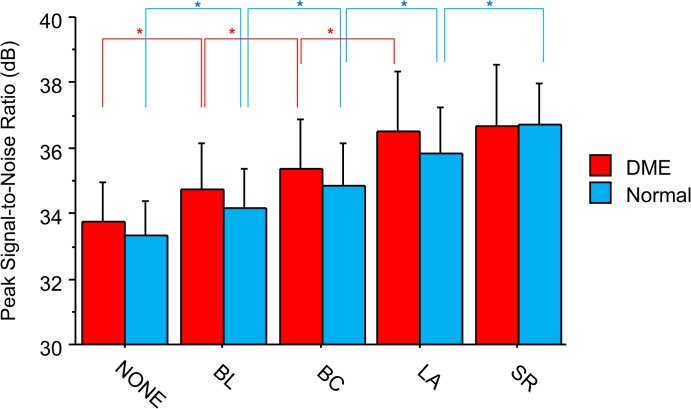
Peak signal-to-noise ratio (PSNR) for each type of recovered optical coherence tomography (OCT) image. Interpolation algorithms examined included the bilinear (BL), bicubic (BC), and Lanczos3 (LA). The super-resolution (SR) algorithm was also investigated. Evaluated OCT images were obtained from normal eyes and from eyes with diabetic macular edema (DME). The higher the PSNR, the closer the degraded image was to the original. The human eye can detect PSNR differences >0.2 dB. *indicates *P* < 0.05 (paired t-test followed by Bonferroni correction).

The PSNR varied between the various enlarged images and was highest in the SR and LA images, which were not significantly different from each other in eyes with DME. The BC images had the next highest PSNR, followed by the BL and NONE images, all of which were statistically different from each other (Fig 2). These results indicate that higher quality images can be obtained with LA and SR algorithms than with the widely-used BL and BC algorithms.

### Retinal nerve fiber layer thickness measurements in enlarged images from normal subjects

**Fig 3:**
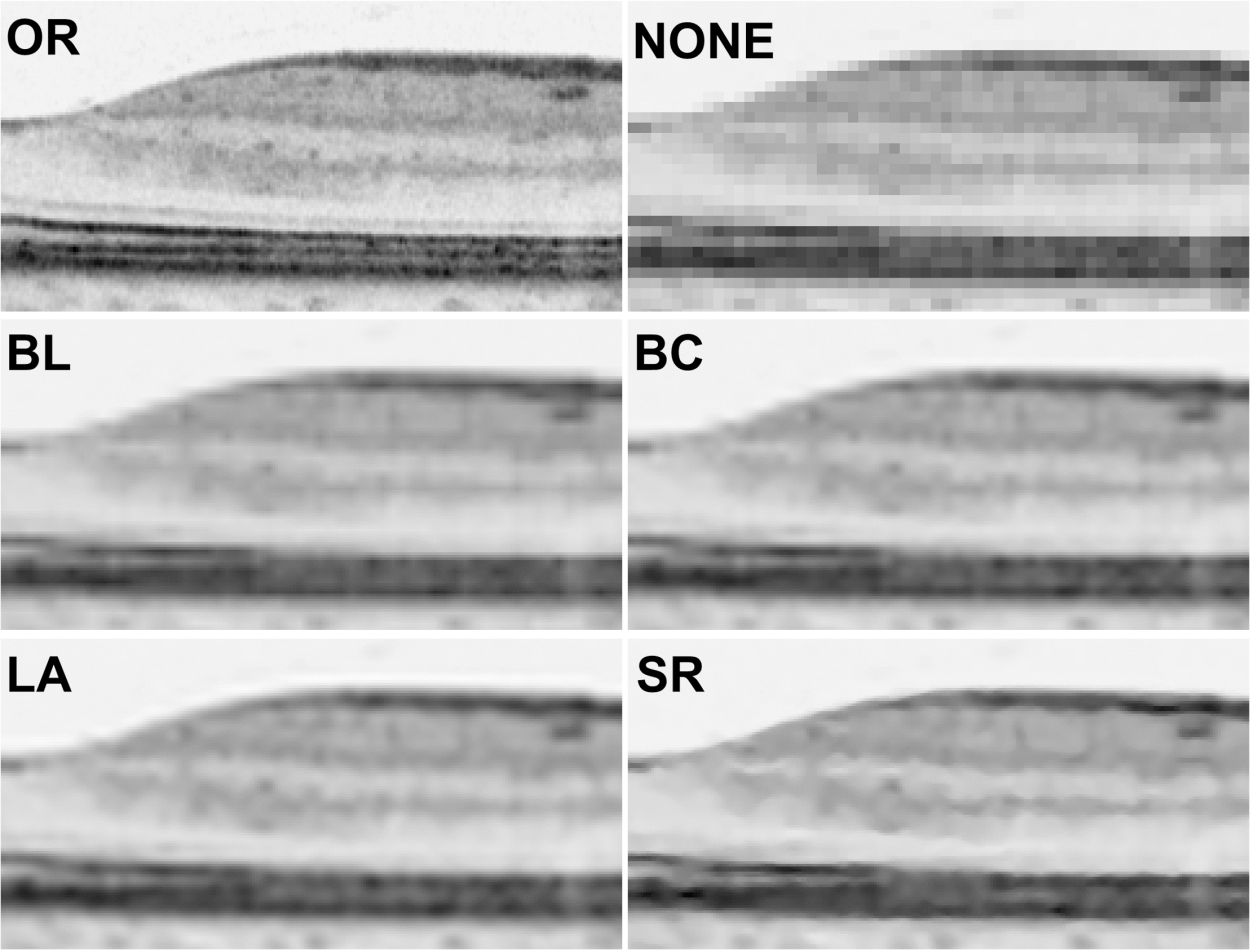
Retinal nerve fiber layer (RNFL) delineation in original and recovered spectral-domain optical coherence tomography images. Images show from the foveal center to 1.5 mm toward the optic disc (horizontal scan) (A) Original image (OR). (B) An enlarged minified image with no additional processing (NONE). The RNFL edge is jagged and is hardly identifiable near the fovea. (C) An enlarged minified image with the bilinear (BL) interpolation algorithm applied. The RNFL edge is smoother than in the NONE image, but the overall image remains blurred. (D) An enlarged minified image with the bicubic (BC) interpolation algorithm applied. The RNFL is less jagged than in the BL image. (E) An enlarged minified image with the Lanczos3 (LA) interpolation algorithm applied. The RNFL edge is smoother than the BL or BC images, with the most remarkable improvement near the foveal pit. However, the image still has a blurred edge. (F) An enlarged minified image with a learning-based super resolution (SR) algorithm applied. The RNFL edge has the best demarcation of all the magnified images, allowing the fovea to be as easily identified as in the OR image. However, texture details and tiny structures (e.g., capillaries) visible in the OR image are not fully depicted.

Interobserver agreement for RNFL thickness measurements was good (ICC = 0.870, *P* < 0.001). Images from each of the six groups are shown in Fig 3. The enlarged image that had no processing (NONE) should have had the same information as the OR image that had been minified by a factor of 4. However, the enlarged NONE image had a jagged RNFL edge, so that the RNFL near the fovea was barely identifiable compared to the OR. Enlarged BL and BC images had smoother edges and were more detailed than the NONE images. However, the images were still more blurry than the OR, but the BC image seemed to be less jagged than the BL image. The LA image had smoother edges than both the BL and BC images, with remarkable differences in RNFL slope towards the foveal pit. The SR image had the clearest RNFL demarcation, in which the RNFL near the fovea was as easily identifiable as the OR image. However, tiny structures (e.g., capillary sections) were not fully depicted.

**Fig 4:**
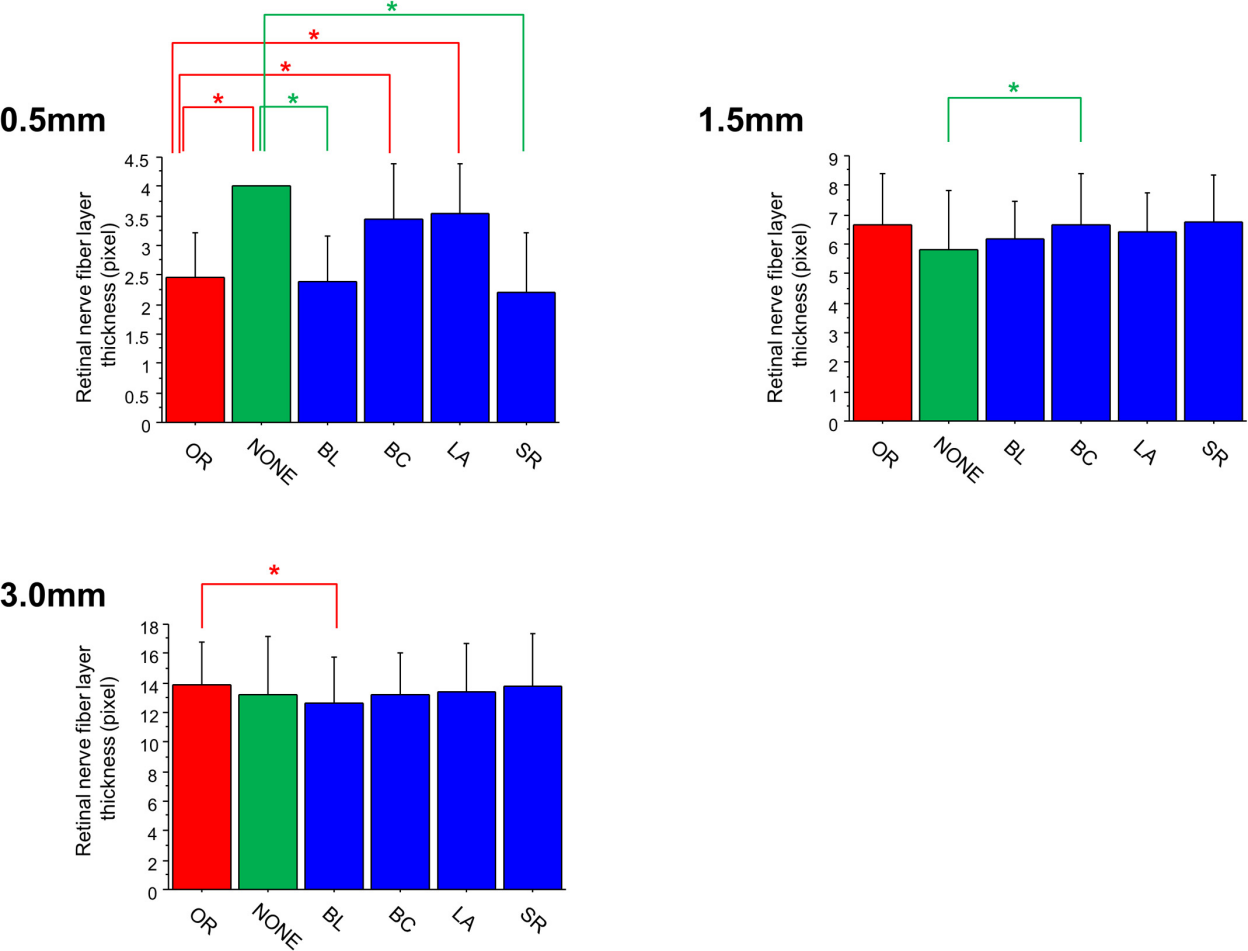
Retinal nerve fiber layer (RNFL) thickness measurements in normal subjects using different image processing techniques. (A) The RNFL thickness measurements 0.5 mm from the fovea. Images recovered without interpolation (NONE), with the bicubic (BC) interpolation algorithm, and with the Lanczos3 (LA) interpolation algorithm resulted in significantly higher measurements than original images (OR). Measurements made on OR images and those recovered with the bilinear (BL) interpolation algorithm or the super-resolution (SR) algorithm were not statistically different. On the other hand, BL and SR images resulted in significantly thinner RNFL thickness measurements than in NONE images. (B) The RNFL thickness measurements 1.5 mm from the fovea. The BC images resulted in significantly thicker measurements than in NONE images. (C) The RNFL thickness measurements 3.0 mm from the fovea. The BL images resulted in significantly thinner RNFL measurements than in the OR images. Similar results were obtained 1.5 mm and 3.0 mm from the fovea. *indicates *P* < 0.05, paired t-test followed by Bonferroni correction.

The RNFL thickness measurements made on each type of image are shown in Fig 4. Measurements made 1.5 and 3.0 mm from the fovea were similar among all image groups, with a few exceptions. Significant differences were noted between measurements made on the NONE and BC images at 1.5 mm (*P* = 0.0034) and between the OR and BL images at 3.0 mm (*P* = 0.0007). However, this was not the case in measurements made only 0.5 mm from the fovea, where multiple differences were detected between measurements made on OR images and those made on processed images. Many differences were also detected between measurements made on NONE images and those made on processed images. However, there were no significant differences in RNFL measurements made 0.5 mm from the fovea between OR and BL images (*P* = 0.8252) or between OR and SR images (*P* = 0.3299). In contrast, NONE, BC, and LA images resulted in significantly higher measurements than OR images (*P* < 0.0001, *P* = 0.0002, and *P* < 0.0001, respectively).

### Qualitative photoreceptor layer assessments in original and recovered images in eyes with diabetic macular edema

**Fig 5:**
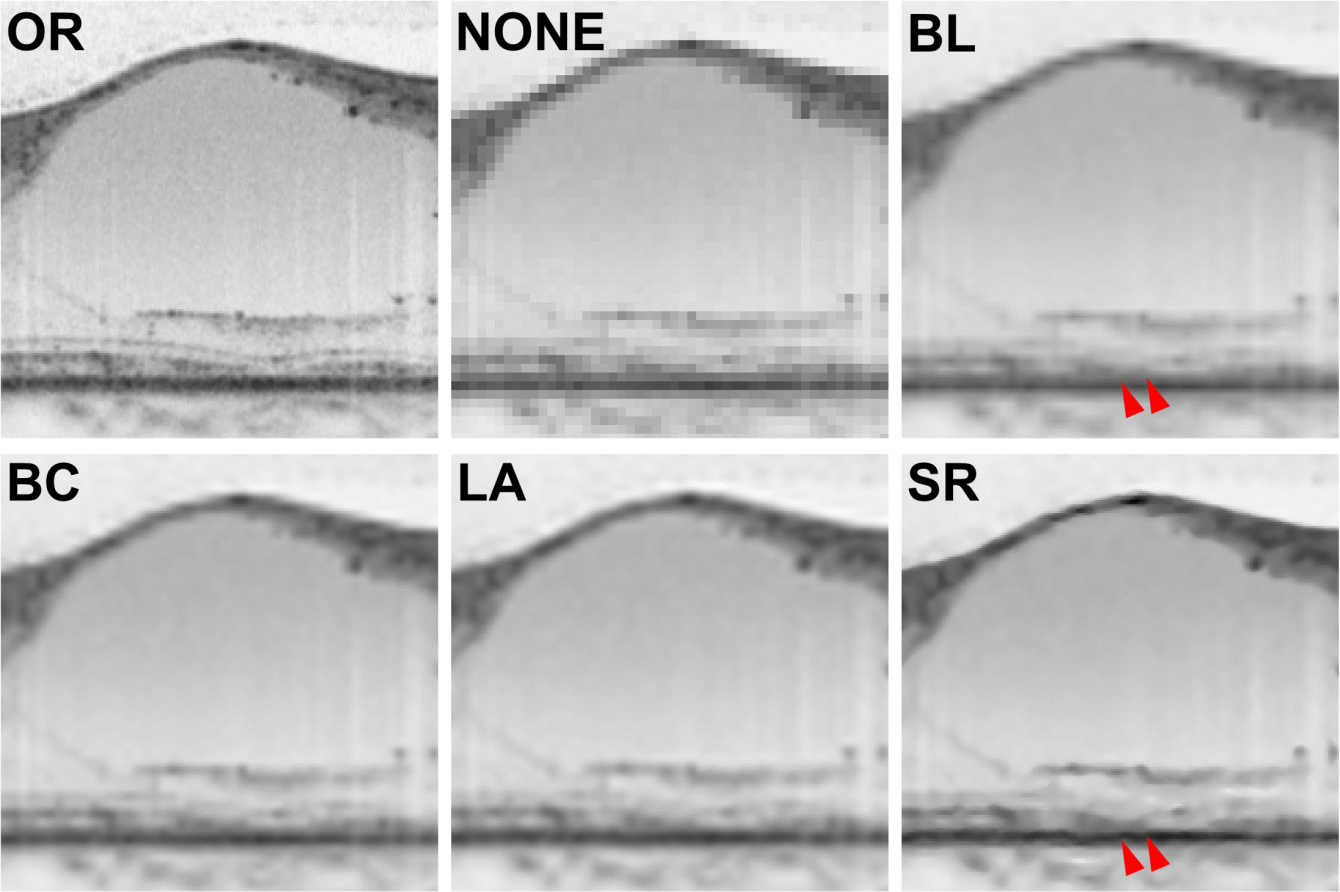
Photoreceptor layer visualization in spectral-domain optical coherence tomography images of eyes with diabetic macular edema. Images show a 1.0 mm horizontal scan centered on the presumed fovea. (A) Original image (OR). Photoreceptor layer status was graded as having a complete external limiting membrane (ELM) line and a discontinuous inner segment ellipsoid line (ISe). (B) An enlarged minified image with no processing (NONE). Aliasing occurred and the ELM and ISe band, which were hardly identifiable, were graded as absent. (C) An enlarged minified image with the bilinear (BL) interpolation algorithm applied. Recovered visualization of the ELM and ISe was achieved. However, an unexpected connection of the ISe band and the ELM (arrowheads) occurred. This did not exist in the OR image. Both the ELM and ISe band were graded as discontinuous. (D) An enlarged minified image with the bicubic (BC) interpolation algorithm applied. Both the ELM and ISe band were graded as discontinuous. (E) An enlarged minified image with the Lanczos3 (LA) interpolation algorithm applied. The RNFL edge is smoother than in the BL and BC images, but the rest of the image does not greatly differ. Both the ELM and ISe band were graded as discontinuous. (F) An enlarged minified image with the learning-based super-resolution (SR) algorithm applied. Although the image has a well demarcated RNFL layer, photoreceptor layer details remain absent. An unexpected connection between the ISe band and ELM is visible (arrowheads).

The Kappa coefficient was 0.757 (*P* < 0.001) for the ISe band and 0.863 (*P* < 0.001) for the ELM, indicating good inter-observer agreement. Representative OCT images of eyes with DME are shown for each of the six image processing groups in Fig 5. Surprisingly, the ISe band and the ELM, which were barely visible in NONE images, became detectable in images recovered with interpolation or SR algorithms. However, the ISe and ELM status in recovered image groups was assessed as more undetectable than in OR images (Tables 1 and 2). Interestingly, none of the recovered images were graded with a complete ISe, but NONE images were graded with an absent ISe or ELM.

**Table 1:** Differences in inner segment ellipsoid line status between original image and recovered images in optical coherence tomographic images of diabetic macular edema.

	Original image			
	Complete	Discontinuous	Absent	*P* Value
NONE				
Complete	0	0	0	
Discontinuous	0	0	0	
Absent	3	23	4	-
BL				
Complete	0	0	0	
Discontinuous	3	13	0	
Absent	0	10	4	0.0261
BC				
Complete	0	0	0	
Discontinuous	3	15	0	
Absent	0	8	4	0.0161
LA				
Complete	0	0	0	
Discontinuous	3	16	1	
Absent	0	7	3	0.0948
SR				
Complete	0	0	0	
Discontinuous	3	14	0	
Absent	0	9	4	0.0214

NONE = image recovered without interpolation; BL = bilinear; BC = bicubic; LA = lanczos3; SR = super-resolution.

**Table 2:** Differences in external limiting membrane line status edema between original image and recovered images in optical coherence tomographic images of diabetic macular edema.

	Original image			
	Complete	Discontinuous	Absent	*P* Value
NONE				
Complete	0	0	0	
Discontinuous	0	0	0	
Absent	14	14	2	-
BL				
Complete	0	0	0	
Discontinuous	9	4	0	
Absent	5	10	2	0.0715
BC				
Complete	1	0	0	
Discontinuous	11	9	0	
Absent	2	5	2	0.1182
LA				
Complete	1	0	0	
Discontinuous	13	7	1	
Absent	0	7	1	0.0387
SR				
Complete	0	0	0	
Discontinuous	11	8	0	
Absent	3	6	2	0.0787

NONE = image recovered without interpolation; BL = bilinear; BC = bicubic; LA = lanczos3; SR = super-resolution.

### Parallelism among the six different imaging groups in eyes with diabetic macular edema

**Fig 6:**
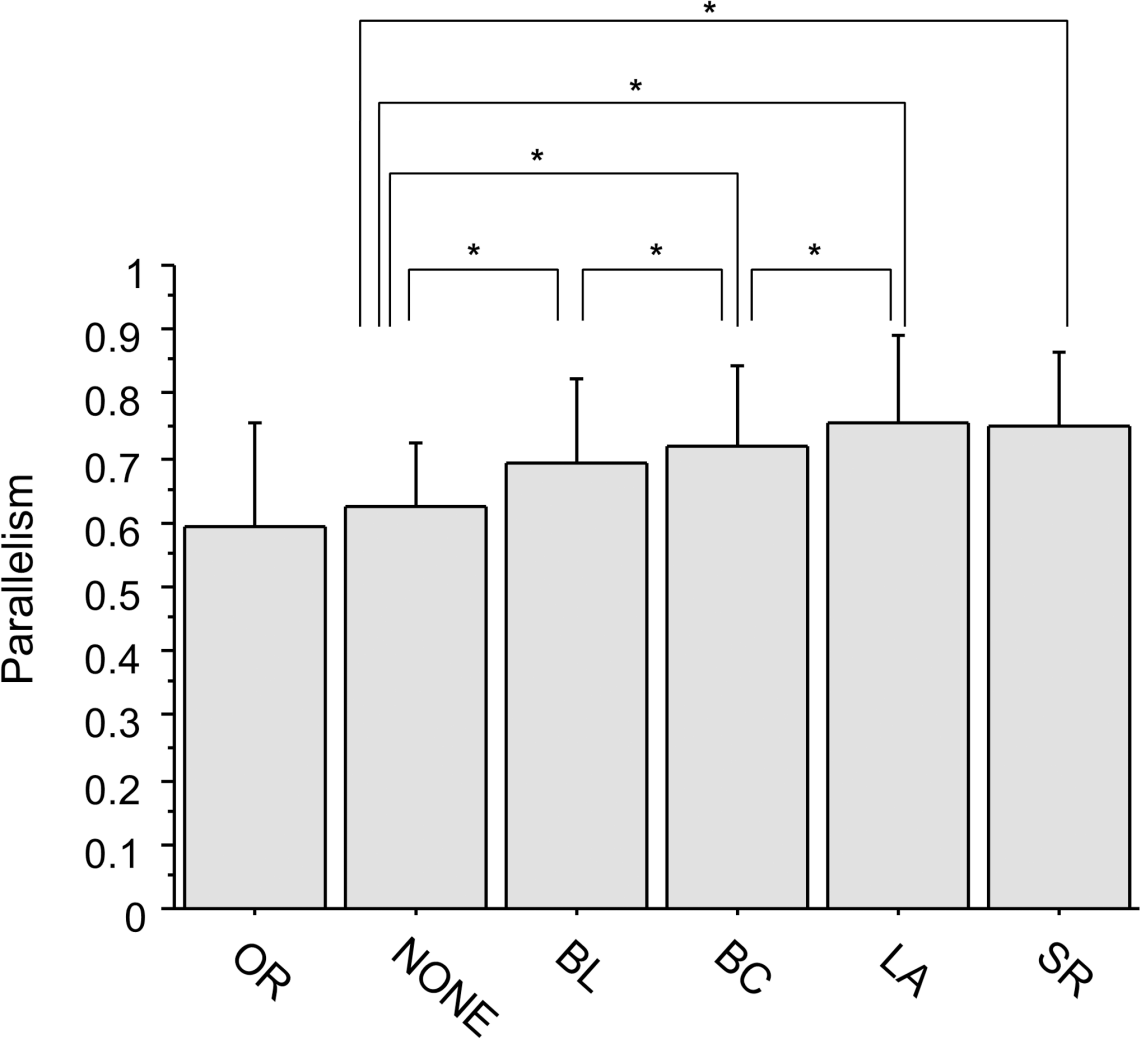
Parallelism of the photoreceptor-retinal pigment epithelium complex in spectral domain optical coherence tomography images in eyes with diabetic macular edema. Parallelism reflects image complexity, which is used for quantitative evaluation of photoreceptor layer structural changes. Parallelism ranges from 0 to 1, with a more complicated, destructive changes resulting in higher parallelism values. Images processed with higher grade algorithms (e.g., super-resolution [SR], Lanczos3 [LA]) tended to have higher levels of parallelism. Additionally, all images processed with enhancing algorithms had significantly higher parallelism than the original (OR) and unprocessed (NONE) images, which were not statistically different from each other. This indicates that photoreceptor layer images were less complicated in processed images than in OR images. BL = bilinear, BC = bicubic.*indicates *P* < 0.05, paired t-test followed by Bonferroni correction.

Quantitative analyses of photoreceptor layer alterations showed that images processed with higher grade algorithms tended to have a higher level of parallelism, which was significantly different between NONE and BL images (*P* < 0.0001), BL and BC images (*P* < 0.0001), and BC and LA images (*P* < 0.0001, Fig 6). The parallelism was not significantly different between LA and SR images (*P* = 0.5667) or between OR and NONE images (*P* = 0.1185), even though the ISe band and ELM were not detectable in qualitative assessment of NONE image. Images from other processing groups, including BL, BC, LA, and SR, had significantly higher parallelism than OR images (all *P* < 0.0001), indicating that photoreceptor layer images in these groups were less complicated than in OR images.

## Discussion

The aim of the current study was to investigate the effect of interpolation algorithms and the SR algorithm on quantitative and qualitative assessments of enlarged images. Study results demonstrated that these algorithms can increase the amount of information on OCT images, even though image recovery was not perfect in any algorithm examined.

Our close examination of smoothing algorithms showed both their usefulness and limitations in OCT image analyses. The PSNR for BL, BC, LA, and SR images was higher than in NONE images, suggesting that these algorithms can increase OCT resolution. Despite this increase, image processing with the BC and LA algorithms can results in larger RNFL thickness measurements 0.5 mm from the fovea than those made on OR images. This difference is thought to result from the blurred RNFL edge in BC and LA images. This finding is important because the BC algorithm is the most widely used.

The SR images had sharper edges than images processed by interpolation algorithms (i.e., BL, BC, and LA), and RNFL thickness measurements in SR and OR images were not significantly different. We believe that the BL algorithm, which also caused a blurred RNFL edge, resulted in thinner RNFL evaluations than the BC and LA algorithms because BL images were less bright and had much more aliasing. Qualitative assessments of the photoreceptor layer in eyes with DME also suggest that image enlargement and reconstruction was helpful. However, the photoreceptor layer in recovered images was less detectable than in OR images, which suggests that reconstructions are not perfect. Furthermore, parallelism, which reflects image complexity, was lowest in the OR image. This implies that interpolation and SR algorithms are not able to recover texture details in OCT images. Together, all these factors tell us that physicians should use caution when evaluating small objects on enlarged and enhanced OCT images. Additionally, clinicians should remember that interpolation algorithms may induce retinal thickness measurement errors.

The amount of information gained from using enhancing algorithms on enlarged images should be considered. Both qualitative and quantitative assessments on enlarged, enhanced (interpolation or SR algorithm applied) images were different from those on enlarged, unenhanced (NONE) images. This suggests that these algorithms changed information in the images. Although increasingly smaller OCT image characteristics are gaining clinicians’ interest,[6,10,11,27] assessment of small objects on enlarged OCT images requires careful attention and consideration. Image enhancing algorithms spuriously increase OCT resolution, which can lead to the creation of image features that did not exist in the original image. Some may even be smaller than one pixel in the original image.

In this study, the SR technique was applied to OCT images. To the best of our knowledge, this is the first paper to examine the effect of this algorithm on OCT B-scan images. The SR method is quite different than interpolation algorithms and is specifically used to recover high resolution images from one or more low resolution images.[12] The SR method can be divided into two main categories, multi-image SR and learning-based SR.[13,16] Multi-image SR combines subtly misaligned (by <1 pixel) images to gain more data points and increase image resolution. This allows very small objects to be depicted in fine detail, however multiple image video recordings are required. Alternatively, learning-based SR can be performed on single images and was used in the current study. Our results showed that SR image enhancement resulted in the highest PSNR and retinal thickness equivalent of OR images. This implies that SR has the potential to allow clinicians to more accurately delineate fine differences in retinal layer thickness. However, SR techniques involve the use of estimation and there is a general tendency to shy away from using such techniques in healthcare. We stress that caution should be used when applying SR methods to OCT images in future studies. The main limitation of our study was the use of minified OCT images because the knowledge obtained here cannot be simply applied to original size OCT images.

## Conclusions

Interpolation and SR algorithms increase OCT image resolution, but qualitative and quantitative image assessment results were influenced by these algorithms. Interestingly, each type of algorithm caused different changes in the assessments. Thus, although enhancing algorithms play a role in OCT image interpretation, evaluation of smaller objects that cannot be evaluated without image enlargement should be done with caution.
